# Multiscale Electron Microscopy for the Study of Viral Replication Organelles

**DOI:** 10.3390/v13020197

**Published:** 2021-01-28

**Authors:** Georg Wolff, Montserrat Bárcena

**Affiliations:** Section Electron Microscopy, Department of Cell and Chemical Biology, Leiden University Medical Center, 2300 RC Leiden, The Netherlands; g.wolff@lumc.nl

**Keywords:** positive-strand RNA viruses, virus–host interaction, electron tomography, blockface imaging, volume SEM, in situ cryotomography, double-membrane vesicles, invaginated spherules, coronaviruses, nodaviruses

## Abstract

During infection with positive-strand RNA viruses, viral RNA synthesis associates with modified intracellular membranes that form unique and captivating structures in the cytoplasm of the infected cell. These viral replication organelles (ROs) play a key role in the replicative cycle of important human pathogens like coronaviruses, enteroviruses, or flaviviruses. From their discovery to date, progress in our understanding of viral ROs has closely followed new developments in electron microscopy (EM). This review gives a chronological account of this progress and an introduction to the different EM techniques that enabled it. With an ample repertoire of imaging modalities, EM is nowadays a versatile technique that provides structural and functional information at a wide range of scales. Together with well-established approaches like electron tomography or labeling methods, we examine more recent developments, such as volume scanning electron microscopy (SEM) and in situ cryotomography, which are only beginning to be applied to the study of viral ROs. We also highlight the first cryotomography analyses of viral ROs, which have led to the discovery of macromolecular complexes that may serve as RO channels that control the export of newly-made viral RNA. These studies are key first steps towards elucidating the macromolecular complexity of viral ROs.

## 1. Introduction

The histories of electron microscopy (EM) and virus research have been closely intertwined since the invention of the electron microscope almost a century ago [1,2]. With a resolution ~100 higher than light microscopy, EM enabled the visualization of the previously invisible virus particles, revealed their elegant structures, and nowadays continues providing essential structural virology information [3]. EM is also a crucial tool to elucidate how viruses replicate in the microscopic battlefield of the infected cell in order to produce new virus particles that lead to infection spread and disease.

**Figure 1:**
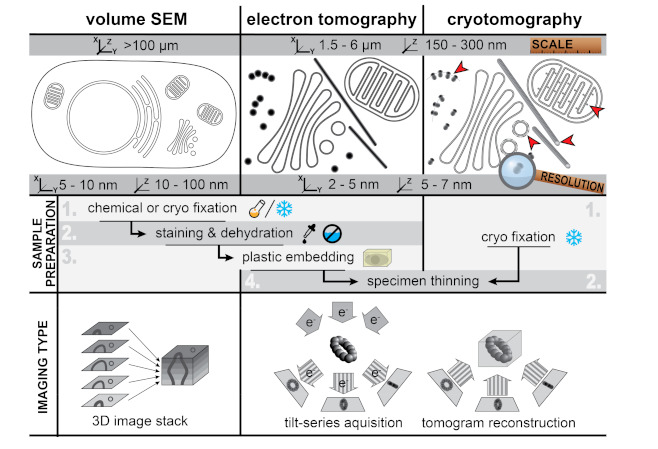
Characteristics of different three-dimensional (3D)-electron microscopy (EM) techniques Figure 3. D imaging of cells at different scales and resolutions: (**left**) volume scanning electron microscopy (SEM) for the imaging of large samples like whole cells and tissue, (**middle**) electron tomography (ET) of resin-embedded samples to analyze subcellular ultrastructure of specific areas in the cell, and (**right**) cryotomography to image the biological content in a near-native state. Volume SEM and ET images of resin-embedded samples have high contrast due to the heavy-metal stain used in sample preparation that accumulates on membranes and large macromolecular features. In cryotomography the contrast is much lower as it is only generated by the biological material itself, which mainly consists of low atomic number elements. However, cryotomography allows the direct visualization of macromolecular complexes like ribosomes and polysomes, clathrin coats, ATP synthases, and individual tubulin subunits in microtubules (red arrowheads). Volume SEM and ET can be applied to resin-embedded samples prepared in a similar manner. For transmission EM (TEM)-based methods (i.e., ET and cryotomography), the samples have to be thinned to a thickness of 100–300 nm prior to imaging. SEM- and TEM-based approaches apply different principles to obtain 3D reconstructions of the specimen. In volume SEM, serial 2D images through the sample are combined to obtain a 3D stack. These 2D images are obtained, for example, by repeatedly imaging the sample blockface after removal of a thin top layer in the material. While the lateral resolution is determined by the diameter of the scanning electron beam, the z-resolution is ultimately limited by the thickness of the layers removed. For electron (cryo)tomography, the specimen is gradually tilted in a TEM to generate a so-called tilt series that consists of 2D projection images of the specimen at different angles. These projections are computationally aligned and combined into a tomographic volume. Due to the geometry of the sample holder and of the own sample (cell sections with slab geometry), the tilt angle is restricted to a maximum of ~70°. This incomplete angular coverage creates an effect, called the “missing wedge”, which results in lower resolution in the z-direction [5].

Contemporary EM encompasses a variety of techniques, covering a wide range of scales, from tissue to macromolecules. Methods to obtain three-dimensional (3D) information at these different scales have been developed (Figure 1). In general, EM techniques can be roughly divided into two main modalities—transmission EM (TEM) and scanning EM (SEM), which each require differently designed electron microscopes [4]. In TEM, an electron beam at least as wide as the field of view penetrates the sample and the electrons transmitted through the sample are used to create a projection image of the specimen that is captured by a post-specimen detector. TEM samples need to be relatively thin (~300 nm at most), due to the limited penetration depth of the electron beam. SEM, in contrast, can be applied to larger specimens. Here, a small, focused electron beam scans the surface of the sample. Different types of signals arising from the electron beam-specimen interaction (e.g., secondary electrons or backscattered electrons) can then be collected in a detector situated above the sample. SEM is best known for its applications to the study of the topography of bulk specimens, such as small organisms, tissues, or cells. However, SEM can also be applied to study intracellular structures, something that has been greatly facilitated by recent technical developments (Figure 1, Section 4). The resolution of SEM is lower than that of TEM, but SEM is particularly well suited to scan large samples or fields of view. Therefore, TEM is the method of choice to study small structural details, whereas SEM approaches help to expand the sample scale.

This review presents an overview of classic and novel EM approaches particularly appropriate for the study of virus-infected cells. We showcase the EM potential with its contribution to the study of viral replication organelles (ROs), a hallmark of positive-strand RNA (+RNA) virus infection in eukaryotes. Viruses as relevant and diverse as coronaviruses, enteroviruses, or flaviviruses drastically remodel the cellular landscape transforming intracellular membranes into distinct structures that support the synthesis of viral RNA. These virus-induced membrane structures have received many names in the literature, from viral factories to replication complexes, but perhaps the term replication organelle (RO) best conveys the idea of a subcellular structure that serves a specific function. The formation of these viral ROs reflects a shared replication strategy that may confer a number of advantages derived from compartmentalization. ROs could provide optimal micro-environments where relevant factors for viral replication concentrate, thus making the process more efficient. Additionally, they could shield replication intermediates like double-stranded RNA (dsRNA) from innate immune sensing. Although many questions about the biogenesis and function of viral ROs remain open, significant progress has been made in the last decades, often going hand in hand with new developments in the field of EM.

## 2. Viral ROs by Conventional 2D and 3D Transmission Electron Microscopy

Cells infected with +RNA viruses started to be analyzed in the second half of last century, shortly after sample preparation methods to investigate cells by TEM developed and matured [2]. It was early recognized that infection with some +RNA viruses resulted in the proliferation of anomalous membrane structures [6,7,8,9]. At the time, however, the function of these virus-induced membrane structures was obscure and they were often described as mere cytopathic effects.

Preserving the fine cellular ultrastructure for TEM studies was not a trivial task and required a number of developments. Cells need to be fixed, stained with heavy metals to generate contrast, gently dehydrated, embedded in a resin, and sectioned. Early sample preparation protocols, which are still widely used nowadays, fixed the cells chemically, typically with aldehyde-based cross-linkers. An alternative pathway is high-pressure freezing (HPF), a method that was first developed in 1968 [10] but only started to spread in the last three decades. In this approach, cells are quickly frozen under high pressure, which hampers the formation of ice crystals that would disrupt the cellular ultrastructure [11]. Water is then gradually replaced by a stain-containing organic solvent as the temperature is slowly increased, a process known as freeze substitution (FS). This is followed by embedding and sectioning of the samples. The tandem HPF–FS is widely considered the gold standard for conventional (i.e., non-cryogenic) EM samples. HPF–FS results in an improved overall morphology [11], even when applied to samples that are chemically fixed in advance (e.g., for biosafety considerations) [12]. This superior ultrastructural preservation is particularly apparent in less distorted membrane structures [11,13].

The analysis of resin-embedded cell sections was initially limited to the 2D projection images that TEM provides. However, at the turn of the century, electron tomography was maturing into an accessible method to reconstruct the 3D structure from 2D TEM images (Figure 1). Soon the first analyses revealing the detailed 3D architecture of virus-induced ROs were published [14,15,16], and the number of tomographic studies of viral ROs has rapidly grown ever since.

**Figure 2:**
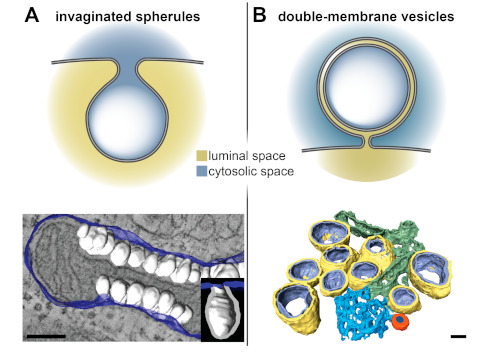
Invaginated spherules and double-membrane vesicles (DMVs) by electron tomography. +RNA virus-induced replication organelles (ROs) can be divided in two classes—invaginated spherules and elaborate vesicular networks that contain DMVs. (**A**) Nodavirus- and (**B**) coronavirus-induced membrane modifications are representatives of these two classes of ROs. (**Top**) Sketches of the architecture of the invaginated spherules and DMVs induced by these viruses. While the invaginated spherules induced by nodaviruses and other +RNA viruses have neck-like openings that connect their interior with the cytosol, the DMVs induced by coronaviruses appear to define close compartments. (**Bottom**) Segmented tomographic volumes of the membrane alterations induced by the nodavirus flock house virus (FHV) in *Drosophila* cells and by the Middle East respiratory syndrome coronavirus (MERS-CoV) in Huh7 cells (adapted from [14] and [39], respectively). FHV induced the formation of invaginated spherules (white) in the outer mitochondrial membranes (blue). Coronaviruses ROs include DMVs (outer and inner membranes in yellow and purple, respectively), convoluted membranes (blue), and double-membrane spherules (orange). These coronavirus-induced membrane structures are often interconnected, either directly or through the ER from which they derive. Scale bars, 100 nm.

With the knowledge that accumulated over years of 2D and 3D TEM, a clear pattern for the classification of viral ROs into two major groups has emerged (Figure 2). Some +RNA viruses induce the formation of invaginated spherules in the membranes of a variety of organelles including mitochondria (nodaviruses [14]), endoplasmic reticulum (flaviviruses [16,17,18,19], bromoviruses [20], tombusviruses [21,22]), and endolysosomes (alphaviruses [23,24,25] and Rubella virus [26]), or in the plasma membrane (alphaviruses [25,27]). Despite their different origins, these viral-induced spherules share the same architecture—they define a small pocket with a neck-like opening that would allow import and export of material with the cytosol (Figure 2A). The second type of RO consists of large and elaborate vesiculotubular networks that seem to derive from membranes of the secretory pathway, most often from the endoplasmic reticulum (ER). These ROs are induced by e.g., picornaviruses [28,29,30,31], noroviruses [32,33], hepatitis C virus [34], arteriviruses [35,36], or coronaviruses [15,37,38,39,40]. The level of complexity of these ROs makes it difficult to relate structure and function in a straightforward manner. These ROs include a variety of virus-induced membrane structures, such as single-membrane vesicles or tubules, paired membrane structures, and multimembrane vesicles (reviewed in [41]), which are specific to the virus group and often also to the stage of infection. Notably, however, these ROs have one element in common—they all include double-membrane vesicles (DMVs) (Figure 2B).

Electron tomography has shown that virus-induced DMVs have variable detailed morphologies. Some establish narrow connections through their outer membrane to other virus-induced structures and/or the endoplasmic reticulum (e.g., coronavirus- or arterivirus-induced DMVs), while others seem to be non-connected independent compartments (e.g., picornaviruses). The interior of the DMVs appears as the kind of optimal secluded environment for viral replication, but a mechanism to exchange material with the cytosol, like the neck-like opening in the invaginated spherules, would be necessary. In this regard, coronavirus-induced DMVs have been particularly intriguing (Figure 2B). For many other viruses, a significant proportion of the DMVs generated in infection have openings that connect their lumen with the cytosol. In contrast, the DMVs induced by coronaviruses and by the distantly-related arteriviruses (both in the order *Nidovirales*) have been characterized as closed compartments in conventional TEM samples [15,36,38,39]. Rare examples of open DMVs have been detected in arterivirus-infected cells [42] and, recently, in cells infected with severe acute respiratory syndrome coronavirus 2 (SARS-CoV-2) [40], but their low frequency suggests that they likely represent short-lived intermediates in the formation of the typically closed nidoviral DMVs.

## 3. Localization of Molecular Factors by Labeling

Mapping relevant molecular players to specific subcellular components is crucial to elucidate the organization of the different steps in the viral replication cycle. Although this can be explored with fluorescence microscopy studying the co-localization between viral and host markers characteristic of a certain organelle, EM offers a number of advantages to consider. In EM images, all the different subcellular structures are directly visualized at nm resolution, thus providing unique contextual information. Fluorescence microscopy, on the other hand, facilitates the rapid localization of rare features of interest. Moreover, using live-cell imaging, key information about the dynamics of the process under study can be obtained. The advantages of each microscopy modality can be synergistically combined using correlative light and electron microscopy (CLEM) approaches. In CLEM, the same exact specimen is analyzed both by light and electron microscopy. CLEM is increasingly used to study viral infection (reviewed in [43]), for example, using fluorescence microscopy to identify positive cells or to capture transient events and small regions of interest that are subsequently ultrastructurally analyzed by EM.

The localization precision in CLEM is ultimately limited by the resolution of light microscopy. However, these limits can be overcome by several EM labeling methods. The most common approach is to use specific antibodies against the molecule of interest. This is the basis of immunoelectron microscopy (IEM). Typically, the location of the antigen is made apparent by an electron-dense colloidal gold particle (5–15 nm) conjugated to a secondary antibody, or to protein A or protein G, which bind to a primary or secondary antibody. In most applications, sections of cells already processed for EM are incubated with the antibody. Therefore, for IEM to succeed, it is essential to preserve the antigen during sample preparation. IEM is compatible with a multitude of resin-embedded approaches, from chemical fixation to HPF-FS [44]. An alternative and commonly used method that was specifically developed for IEM is the Tokuyasu technique, in which immunogold labeling is performed on thawed cryosections [45]. A general consideration is that antigenicity is easily lost with the high concentrations of stains and fixatives that would provide the best contrast and ultrastructural preservation. Some compromise is inevitable and finding the right balance between effective labeling and recognizable ultrastructural motifs is often the bottleneck of IEM.

IEM provided some of the first clues about the role of the membrane structures induced in infections with +RNA viruses. Viral non-structural proteins were shown to localize to these structures [20,24,25,28,35,37,46], an observation that has been consistently reported for multiple +RNA viruses since these initial studies. Collectively, the viral non-structural proteins ensure viral replication by executing a variety of functions, from inducing the formation of viral ROs (reviewed in [41,47]) to carrying out enzymatic functions associated with the synthesis of viral RNAs. Negative-sense viral RNAs are first synthesized to serve as template for new copies of positive-strand RNAs, and dsRNA intermediates are generated in the process. Similarly to many non-structural proteins, dsRNA has also been shown to localize in the virus-induced membrane structures for several +RNA viruses [15,16,21,32,36,39]. Together, these results suggest that the virus-induced membrane structures accommodate viral RNA synthesis, thus serving as viral ROs. It is worth noting, however, that an intrinsic limitation of this type of analysis is that it is not possible to ascertain whether the detected non-structural proteins and dsRNA are actively involved in viral RNA synthesis. In fact, only a small percentage of these may be engaged in active replication complexes at a given time [48].

Arguably, a more reliable method to localize viral RNA synthesis is to label newly-synthesized viral RNA. To this aim, the infected cells are provided with an RNA precursor that allows subsequent detection by EM. Concurrently, host transcription is arrested (e.g., with actinomycin D) to restrict the metabolic labeling to viral RNA. A common strategy is the IEM detection of bromouridine-5’-triphosphate (Br-UTP) incorporated into viral RNA [14,20,25,30,35,37]. Alternatively, newly-made viral RNA can be radiolabeled using a radioactive precursor (e.g., tritiated uridine) and detected by EM autoradiography [23,31,39,46,49,50]. This technique is very sensitive as it relies on radioactive disintegrations on a photographic emulsion that ultimately results in electron-dense grains that can be detected by EM [4,51]. Its spatial resolution, however, is lower than in IEM where the distance from the signal to the epitope is determined by the size of the bridging antibodies (15–30 nm). In contrast, an autoradiography signal can be a few hundred nanometers away from the radioactive source, due to the random direction of the radioactive disintegrations. Therefore, the interpretation of EM autoradiography samples relies on the (quantitative) analysis of the signal distribution around different subcellular structures, rather than on the precise location of individual grains.

When applied to cells infected with +RNA viruses, both metabolic labeling approaches have clearly established that the virus-induced invaginated spherules do accommodate viral RNA synthesis [14,20,23,25]. For viruses that generate more elaborate ROs including DMVs, it is not in all cases clear whether all the different virus-induced membrane structures or only a subset of them harbor active replication complexes. The results for coronaviruses and picornaviruses, which are the best characterized examples in this regard, illustrate two different scenarios with some common elements.

**Figure 3:**
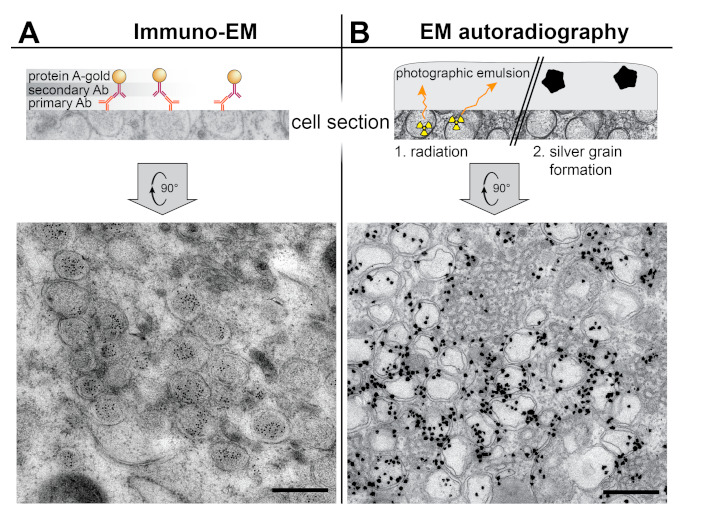
Labeling EM techniques applied to the study of the coronavirus ROs. (**A**) Immunoelectron microscopy (IEM) detection of dsRNA inside the DMVs induced by SARS-CoV in Vero E6 cells (adapted from [15]). On-section immunogold labeling was performed on sections from plunge-frozen freeze-susbstituted cells using a primary antibody (Ab), a secondary antibody, and protein A conjugated with 10 nm gold. (**B**) EM autoradiography detection of active viral RNA synthesis showing it is associated to coronaviral DMVs. Tritiated uridine was provided to live Huh7 cells infected with MERS-CoV, so that the radioactive label could be incorporated into newly-synthesized viral RNA. After 30 min, the cells were chemically fixed, prepared for EM, sectioned, and covered by a thin layer of photographic emulsion. In EM autoradiography, the radioactive disintegrations that arise from the sample in random directions create defects in the emulsion that give rise to electron-dense silver grains upon development. The image shows the high density of label in areas containing MERS-CoV-induced DMV (adapted from [39]). Scale bars, 500 nm.

The site of viral RNA synthesis in coronavirus-infected cells has been particularly controversial, and the efforts to localize it nicely illustrate the potential and limitations of different methods (Figure 3). In IEM samples of SARS-CoV-infected cells, dsRNA was shown to accumulate inside the DMVs [15] (Figure 3A). However, the idea of viral RNA synthesis taking place inside the DMVs was challenged by the systematic characterization of coronaviral DMVs as closed compartments, without an apparent import/export connection to the cytosol. Without formal proof of viral RNA synthesis, DMVs could simply be sites of dsRNA accumulation, perhaps to conceal it from innate immune sensing. Attention thus turned to other coronavirus-induced membrane structures as possible alternative or additional sites of viral RNA synthesis. These include paired ER membranes that often appear in intricate arrangements termed convoluted membranes (CM) [15,52,53,54] and the small and often open double-membrane spherules (DMSs) [38,39,55] (Figure 2B). An early metabolic labeling study using Br-UTP and IEM in cells infected with mouse hepatitis virus (MHV) suggested that viral RNA synthesis associated with coronavirus-induced DMVs [37], However, the DMV morphology was compromised to preserve antigenicity and CM and DMSs could not be detected in these conditions. Recently, these difficulties were overcome in an EM autoradiography study that analyzed cells infected with different coronaviruses [39]. Since this approach does not rely in antigenicity preservation, it was compatible with high-contrast sample preparation methods that distinctly revealed all the different structural motifs in the coronavirus RO. Due to the limited resolution of autoradiography, however, imaging had to be complemented with quantitative analyses. The results clearly demonstrated that DMVs are the primary sites of viral RNA synthesis, and likely the only ones, as no signal could be unambiguously assigned to the other coronavirus-induced structures (Figure 3B) [39].

Viral RNA synthesis is not restricted to DMVs in cells infected with picornaviruses. In fact, active replication has been shown to associate with all picornavirus-induced membrane elements, from the initial single-membrane structures to the DMVs and multilamellar vesicles that proliferate later in infection [30,31,50]. This may relate to the fact that the early single-membrane tubules and vesicles are most likely the precursors of the late multi-membrane compartments [29,30,31]. Interestingly, picornavirus-induced single-membrane vesicles and tubules may be the most relevant RO elements, as they predominate at the peak of viral replication [29,30,31]. Despite these differences, a common factor between coronaviruses and picornaviruses is the use of virus-induced DMVs for viral RNA synthesis. For hepatitis C virus, newly-made viral RNA has also been detected in association with DMVs isolated from infected cells [56]. Therefore, like the invaginated spherules, DMVs appear to be suitable general platforms for viral RNA synthesis. Further analogies and differences across families of viruses that induce vesiculotubular ROs and the possible specific roles of the different virus-induced membrane structures largely remain as open important questions for future research.

## 4. Zooming Out—Volume Scanning Electron Microscopy

Electron tomography is typically used to analyze small regions in the cell. The high magnifications that enable the study of fine ultrastructural details reduce the field of view to a few micrometers. Moreover, the reconstructed depth is limited to the cell section thickness (Figure 1). Larger volumes can be obtained by combining 3D reconstructions from overlapping regions and/or from consecutive cell sections (i.e., serial tomography) [57]. Although in theory this could expand the analyzed regions as desired, in practice, reconstructing whole cells or pieces of tissue in this manner is a monumental task.

**Figure 4:**
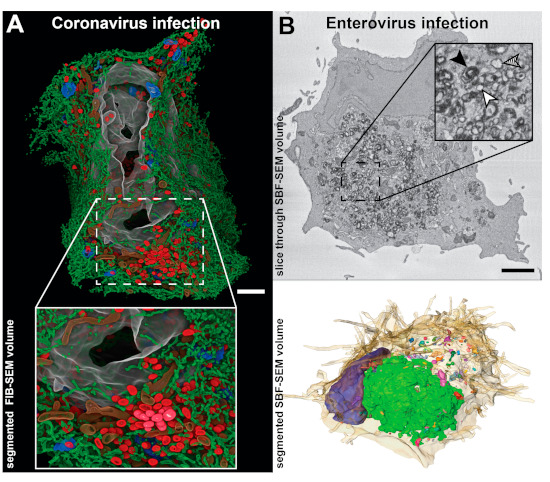
Studies of viral ROs in whole cell 3D SEM reconstructions. (**A**) Focused ion beam SEM (FIB-SEM) segmented volume of a whole human pulmonary epithelial Calu-3 cell at 24 h after infection with SARS-CoV-2 (adapted from [40] with permission). The large-scale data showed how a large network of DMVs (red) and endoplasmic reticulum (ER) (green) spreads throughout the cell, while the resolution was sufficient to resolve small membrane connections between the elements of this network. Other segmented cellular features include Golgi membranes (blue), mitochondria (brown), and nucleus (grey). (**B**) Whole-cell serial blockface SEM (SBF-SEM) of a CVB3-infected Vero E6 cell at 6 h post-infection. (**Top**) A slice through the volume displaying abundant ROs in the perinuclear area of the cell. Individual membrane bilayers could not be resolved in the images due to the limited SEM lateral resolution. Despite this, single-membrane, double-membrane and multilamellar ROs could be distinguished by the thickness of the stained membranes (inset: white, hatched and black arrowheads, respectively). (**Bottom**) Corresponding segmented volume exposing the heterogenous distribution of differently sized RO clusters (multicolored) around the nucleus (blue) and within the cell (beige, semitransparent). Images adapted from [59]. Scale bars, 2 µm.

In recent years, new EM methods based on SEM have been developed to reconstruct large 3D volumes (reviewed in [58]). Here we will highlight two volume SEM approaches that have been applied to the study of viral ROs and are based on a similar principle—serial blockface SEM (SBF-SEM) and focused ion beam SEM (FIB-SEM). In both, stacked images through the volume of a resin-embedded sample are obtained by repeated cycles in which the block surface is first imaged and then removed to expose the next level in the material. The top layer is removed either by a built-in diamond knife (SBF-SEM) or a focused ion beam (FIB-SEM). Overall, the resolution is lower than in TEM approaches (Figure 1), most notably in z, where it is limited by the thickness of the layer removed in each cycle (at least ~25 nm in SBF-SEM and ~5 nm in FIB-SEM). However, the 3D volumes generated can span hundreds of microns in each dimension, enabling the reconstruction of whole cells and pieces of tissue. Volume SEM technology has been applied, for example, to analyze cells infected with coronaviruses [40] and enteroviruses [59] (Figure 4), providing comprehensive pictures of the cellular alterations occurring upon infection.

Since the first ET studies, coronavirus-induced DMVs have been thought to be part of an interconnected membrane network due to the membrane connections that the DMVs established with other DMVs, the ER and alternative virus-induced structures [36]. FIB-SEM reconstructions of human pulmonary epithelial Calu-3 cells infected with SARS-CoV-2 have recently enabled the direct visualization of these large networks of modified ER membranes [40] (Figure 4A). The data revealed different-sized clusters of DMVs that were interconnected by stretches of paired membranes like grapes in a bunch. These clusters were linked to the ER, which is the apparent membrane donor organelle, and together they formed an intricate network throughout the infected cell.

Enteroviruses form a genus in the *Picornaviridae* family that includes human pathogens such as poliovirus, coxsackiviruses, or rhinoviruses. Enterovirus-induced membrane structures include single-membrane tubules that transform into DMVs and multilamellar vesicles as infection progresses. Although electron tomography characterized them as non-interconnected compartments, these structures seemed to cluster together [29,30]. SBF-SEM data of cells infected with coxsackievirus B3 (CVB3) showed that the size and distribution of RO clusters are not homogenous, with most virus-induced structures accumulating in a single cluster in the perinuclear area [59] (Figure 4B). In the same study, a CLEM approach combining live-cell imaging and SBF-SEM was applied to investigate the biogenesis of CVB3-induced ROs. Live-cell imaging was used to monitor the appearance of the first RO foci at early stages in infection. Next, the same cells were analyzed by SBF-SEM, all the emerging ROs were localized in the reconstructed volumes and their association and membrane connections with other cellular organelles analyzed. The study showed that enteroviruses sequentially use ER and Golgi membranes to generate ROs and that lipid droplets are recruited to the emerging RO regions [59]. Contact sites between enteroviral ROs and lipid droplets appear now to be critical to provide access to lipids required for RO formation and viral replication [60,61].

As a new emerging technology, volume SEM remains to be widely applied to the study of viral ROs and, in general, to the study of viral infection. The previous examples, however, illustrate its great potential in addressing questions that require both the whole cellular context and nanometer resolution.

## 5. Zooming In—Cryo-Electron Microscopy

In conventional EM specimens the resolution is ultimately limited by the characteristics of the sample. The fixation, staining, dehydration, and embedding steps introduce elements that are artificial to the originally-live cell. During the process, artifacts may be induced and material may be extracted. Furthermore, in these samples the biological material is only indirectly visualized through the stains that react with certain cellular components. This sets a fundamental limit to the level of detail that can be faithfully interpreted. Resin-embedded samples have excellent contrast and are relatively resistant to the highly ionizing electron beam, which makes them very well suited for the screening of large number of cells and the study of subcellular architecture. However, they fail to reveal the rich macromolecular content of the cell. These limitations can be overcome by cryo-EM.

Cryo-EM, which emerged in the 1980s [62,63], represents a completely different approach to sample preparation. Here, the priority is not to optimize the contrast or the sturdiness of the sample, but to preserve the biological material as close as possible to its native conditions. Logically, this should include maintaining its natural aqueous environment. To this end, the samples are most commonly plunge-frozen into a liquid cryogen (e.g., liquid ethane), so that water quickly transforms into a solid amorphous (i.e., vitreous) state without crystallizing. Once vitrified, the samples must be kept below the water devitrification temperature (~−135 °C) during handling and imaging. Due to the lack of stain, cryo-EM samples have intrinsically low contrast. They are also highly sensitive to radiation damage, which imposes strict low-dose conditions during imaging. This results in noisy images that do not directly provide high-resolution information.

**Figure 5:**
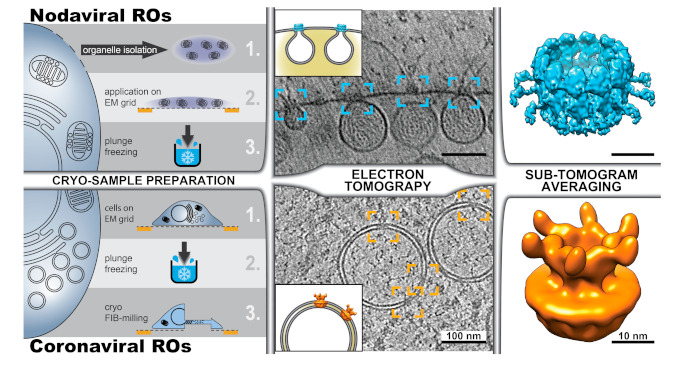
Cryotomography of viral ROs. Different possible workflows are exemplified with the recent cryotomography studies of the ROs induced by (**top**) nodaviruses (FHV) [70,71] and (**bottom**) coronaviruses (MHV) [72]. (**Left**) Cryotomography of subcellular structures can be performed on (**top**) plunge-frozen structures extracted from the cell or (**bottom**) on intact cells. The latter approach, known as in situ cryotomography or cellular cryotomography, requires the thinning of frozen cells, which can be carried out with a focused ion beam that mills away parts of the cell to create a thin cryo-lamella. Cryotomography revealed the presence of crown-like protein complexes in the necks of nodavirus-induced invaginated spherules (blue boxes) and spanning the two membranes of coronavirus-induced DMVs (orange boxes) (tomographic slices adapted from [70] and [72], respectively). For sub-tomogram averaging, hundreds to thousands of sub-volumes containing these crown-shaped complexes were extracted from the cryotomograms, aligned in 3D, and averaged. Following this approach, the structure of the FHV spherule crown complex was resolved at 8.5 Å resolution (top, surface rendering of the Electron Microscopy Data Bank (EMDB) entry EMD-22129, [71]), and the structure of the DMV-spanning MHV pore complex was determined at 30.5 Å (bottom, rendered from EMDB entry EMD-11514, [72]).

Cryo-EM has been widely applied to purified specimens like macromolecular complexes or icosahedral viruses. In these cases, each imaged particle represents a different view of the same structure. Thousands of these individual images can then be combined and averaged to increase the signal-to-noise ratio and obtain a 3D reconstruction. This is the principle of single particle analysis, which nowadays can routinely achieve near-atomic resolution [3,64,65]. For specimens where averaging is not possible, cryotomography can be applied to reconstruct their 3D structure [5] (Figure 1). Specimens in this category include pleomorphic viruses and subcellular structures like viral ROs. While cryotomography has been extensively applied to pleomorphic viruses (for recent examples, see [66,67,68,69]), the applications to viral ROs are only quite recent and limited (Figure 5). In a pioneering study, Ertel et al. analyzed the replication organelles that flock house virus (FHV) induces in the outer membrane of the mitochondrial membrane [70]. To this end, mitochondria were purified from FHV-infected cells, plunge-frozen, and analyzed by cryotomography (Figure 5, top). The invaginated spherules that FHV induces in the outer mitochondrial membrane were shown to be filled with densely packed RNA fibrils, largely in a dsRNA form. Most strikingly, cryotomography revealed the presence of a molecular complex that crowns the neck-like opening in the invaginated spherules and that had not been detected before in conventional EM samples. Using IEM, the authors demonstrated that this complex is formed by protein A, a membrane-associated viral protein that performs all the enzymatic activities required for viral RNA synthesis and capping. Fibrils that likely represented viral RNA exiting the viral RO were often detected in association with the protein A complexes.

Although cryotomography can directly visualize macromolecular components in the sample, the resolution of cryotomograms is relatively modest because of the low dose allowed and the geometry of data collection (Figure 1). However, when multiple copies of the same structure are present in these 3D reconstructions, the same principles of single-particle analysis can be applied. The approach is known as subtomogram averaging [73]. Small sub-volumes (subtomograms) containing copies of the structure of interest are extracted from the tomograms, aligned to each other and averaged. Subtomogram averaging of the protein A complexes in the neck of nodavirus-induced invaginations revealed a dodecameric ring-like assembly at around 3 nm resolution [70]. Recently, with a much larger number of particles, the structure of the complex has been determined at 8.5 Å resolution [71] (Figure 5, top right). At this resolution, the different domains in the central turret and flanking densities of this ~35 nm diameter complex became apparent. In the same study, the N-terminal domains of the protein A subunits were mapped to the apical part facing the cytosol [71].

The ideal framework to study viral infection and viral ROs is the infected cell. In situ cryo-EM not only prevents undesired effects that may be introduced in the purification steps, but also uncovers the cellular context of infection with macromolecular resolution. However, cryo-EM of cells has been traditionally hampered by the cell thickness, which largely exceeds the ~300 nm upper limit required for TEM. Cryo-ultramicrotomy of frozen samples was an early development in cryo-EM [74], but, compared with the embedding resins used in conventional EM, vitreous ice is a much less favorable material for mechanical sectioning. Preparing vitreous cellular sections requires highly-trained skills and artifacts like compression and crevasses are easily induced, when not unavoidable [75]. In the last decade, focused ion beam (FIB) milling has emerged as a useful alternative to prepare thin samples from cells [76,77,78]. The method consists of using a FIB, which is integrated in a scanning electron microscope, to chisel a thin layer in the sample. In most applications, cellular material above and below a region of interest is removed, resulting in a thin slice (100–300 nm), termed a cryo-lamella, that is held by the remainder of the cell (Figure 5, bottom). These cryo-lamellae are then imaged by TEM and cryotomography is performed in selected areas.

Cellular cryotomography has been recently used to investigate different steps in the coronavirus replication cycle and newly-formed coronavirus particles in situ [72,79]. The study by Wolff et al. focused on the analysis of coronavirus-induced ROs and provided an answer to the long-standing question of how newly-made viral RNA, presumably generated inside the coronaviral DMVs, can be exported from these apparently closed compartments for translation and packaging into progeny virions [72]. Cryotomography unveiled crown-shaped macromolecular pores that span the double membrane of the DMVs, thus connecting the DMV lumen with the cytosol (Figure 5, bottom). Subtomogram averaging was applied to solve the structure to ~3 nm resolution, revealing a 6-fold symmetry complex assembled around a central channel (Figure 5, bottom right). In order to avoid the use of fixatives and preserve the samples as close as possible to their native conditions, the study focused on the DMVs induced by mouse hepatitis virus (MHV), a well-established model coronavirus that does not pose serious biosafety constraints for cryo-EM sample preparation and imaging. However, similar DMV-spanning complexes were also detected in pre-fixed cryo-EM samples of cells infected with SARS-CoV-2 [72,79]. This supports the notion that the DMV-spanning molecular pore is a generic coronavirus complex.

Conventional labeling techniques are not compatible with cryo-lamellae and, therefore, identifying specific macromolecules in cellular cryotomograms can be a challenge. However, using a recombinant MHV that expresses a green fluorescent protein (GFP) fused to the N-terminal domain of nsp3 [80], it could be shown that the core of the coronaviral DMV-spanning pore complex is formed by a hexameric assembly of this multi-domain transmembrane protein. An additional mass, compatible with the expected size of GFP, was detected in each of the six prongs of the cytosolic crown of the complex [72]. Other viral or host integral components of this large macromolecular complex (estimated mass: ~3 MDa) remain to be identified and may include nsp4 and nsp6, the other two coronaviral nonstructural proteins with transmembrane domains. In particular, nsp4, which together with nsp3 has a critical role in DMV formation [81,82,83], appears as a likely candidate. Variable densities were frequently detected in association with the luminal and cytosolic sides of the pore complexes, which suggests that additional factors may dynamically interact with the DMV-spanning molecular pore [72]. The identity of these factors is unclear, but an attractive possibility is that these interactions help to coordinate viral RNA synthesis, RNA export, and RNA packaging. Parts of the replication machinery may interact with the complex via its luminal side, possibly to guide newly-made viral RNA through the pore. At the cytosolic side, interactions between nsp3 and the nucleocapsid protein may facilitate packaging of the exported viral RNA.

The analogies between the crown-shaped macromolecular complexes in coronavirus and nodavirus ROs are striking [84], even more so considering the large evolutionary distance between these viruses and the rather different morphology of the replication compartments that they induce. Recently, the structure of the nsP1 protein of Chikungunya virus, which binds to membranes and has viral RNA capping-related activities, has been solved in a dodecameric ring-like complex by cryo-EM and single-particle analysis [85]. The high-resolution structure of this nsP1 assembly strongly suggests that it may be (part of) a molecular gate in the neck of the invaginated spherules generated by this alphavirus [85]. Taken together, these studies suggest that viral complexes mediating the release of viral RNA from the ROs may be an advantageous trait shared by many +RNA viruses. Moreover, due to their likely key role in viral replication, these complexes offer new targets for antiviral interventions. These are interesting possibilities that will undoubtedly be explored in the coming years.

## 6. Concluding Remarks and Outlook

EM continues to be an indispensable tool for the study of viral infection. The current full spectrum of EM modalities covers an extensive range of scales and resolutions. The different EM techniques, each with its own advantages and limitations, provide complementary information. Many of the methods covered in this review, such as electron (cryo)tomography, CLEM, volume SEM, or subtomogram averaging have emerged or matured only in the last 20 years. Like for many other biological systems, our understanding of viral ROs has been advancing thanks to developments in EM. Electron tomography has been instrumental in disclosing the 3D architecture of these fascinating virus-induced structures. Newer developments like volume SEM orcryotomography remain to be fully explored, but the few studies to date on viral ROs illustrate their enormous potential.

Due to its unique capability to provide 3D structural information of large samples at nanometer resolution, volume SEM will be crucial to study viral infection in larger, tissue-wide contexts and to get new insights into viral pathogenesis [86,87,88,89]. Cryotomography, on the other hand, with its macromolecular resolution, will be instrumental to zoom in into the molecular details of viral infection. While this review focused on volume SEM of resin-embedded samples, it is worth mentioning that FIB-SEM can also be applied to frozen specimens [90,91], thus circumventing possible artifacts of conventional EM sample preparation. Importantly, this approach can be integrated in workflows involving other cryo-imaging modalities like cryo-LM and cryotomography [92], which opens the door to new future applications.

In situ cryotomography of FIB-milled cryo-lamellae is an outstanding recent development that bridges the gap between structural and cell biology. However, the fact that only a very small region of a cell (the cryo-lamella) is available for investigation and the difficulties in labelling components in the sample are limitations that are hard to overcome. Other shortcomings related to sample preparation, such as the low-throughput of this highly demanding technique, are likely to be reduced in the near future thanks to on-going technical developments [93,94,95]. The power of cryotomography to shed new light into extensively-characterized biological structures is nicely illustrated by the recent studies of viral ROs. The crown-shaped molecular complexes unveiled in the nodavirus and coronavirus replication compartments have already transformed our view on these virus-induced structures. Viral gates to regulate the transit from/to the ROs and, possibly, to coordinate it with other processes in the viral replication cycle may well be a common theme among +RNA viruses. Verifying this hypothesis, elucidating how these viral complexes work and designing strategies that interfere with their function are exciting and challenging goals for the future.

## Data Availability

All Data generated or analyzed are contained within the present article.
